# Sex-differences in Mountain Ultra-trail Performance: Look at the Scenery

**DOI:** 10.1186/s40798-025-00894-x

**Published:** 2025-07-26

**Authors:** Grégoire P. Millet, Alexa Callovini, Antoine Raberin

**Affiliations:** 1 Institute of Sport Sciences, Faculty of Biology and Medicine, University of Lausanne, Lausanne, 1015 Switzerland; 2 CeRiSM, Sport Mountain and Health Research Centre, University of Verona, Rovereto, Italy

## Abstract

There is a growing body of literature on sex-differences in human performance, particularly in the context of endurance sports. However, several mechanisms (e.g., higher type 1 fibres proportion; lesser neuromuscular fatigue; higher metabolic flexibility etc) have been previously proposed and suggest an advantage to females over ultra-endurance competitions on flat terrain. However, in mountain ultramarathon, the percent sex difference between male and female records appears to be larger than on various road/track running distances on flat terrain, suggesting that additional factors related to the specific mountainous conditions may be at play. In this Current Opinion, we point to three specific factors that are likely to influence and widen sex differences in ultra-distance running events performed in the mountains (i.e., uphill– downhill locomotion, altitude, and changes in extreme ambient temperatures).

First, the sex differences in uphill endurance performance are approximately two times larger than those in events primarily performed on flat terrain (i.e., 18–22% vs. 9–12%, respectively), mainly due to the detrimental influence of the lower lean mass to fat mass ratio and lower fast twitch/type II fibre type proportion in females).

At altitude, the ventilatory response to exercise emerges as one of the most sex-sensitive factors that may modify reactions to hypoxia. A diffusive mechanism appears to be involved in the larger hypoxemia commonly reported in females.

Finally, responses to cold environments are also sex-dependent, with females exhibiting lower muscle mass, which limits thermogenic heat production, a higher body surface area-to-mass ratio, and a greater prevalence of Raynaud’s phenomenon.

Altogether, these specific factors must be further understood when analyzing sex-differences in mountain ultra-trail performance. Don’t forget to look at the scenery!

## Key Points

1. Three specific factors are likely to widen sex differences in ultra-distance running events performed in the mountains, compared to ultra-endurance competitions on flat terrain (i.e., uphill– downhill locomotion, altitude, and changes in extreme ambient temperatures).

2. The sex differences in uphill endurance performance are approximately two times larger than those in events primarily performed on flat terrain.

3. At altitude, a reduced ventilatory response, an increased cost of breathing and a higher prevalence of exercise-induced hypoxemia in females, emerge as factors that may modify sex-differences in endurance performance in hypoxia.

4. Females experience thermoregulatory disadvantages in cold environments, exacerbated by simultaneous high altitude exposure and repeated transitions between cold and hot-dry conditions during mountain ultra-trail races.

## Introduction

There is a growing body of literature on the sex differences in human performance [1, 2]. Overall, males have general characteristics (e.g., larger muscle mass, heart size, lung capacity, bones, strength, and circulating haemoglobin) that are paramount for sports performance and that give them advantages in many domains of human athletic performance [3]. However, it has been proposed that females have a relative competitive advantage (i.e., the difference in performance is narrower) in endurance sports with increasing distances due to several mechanisms (e.g., higher type 1 fibres proportion; lesser neuromuscular fatigue; higher metabolic flexibility etc) [4, 5].

However, despite these well-established factors, the sex differences (when expressed as a percentage of the male record) in ultra-running world records (as of November 1, 2024; iau-ultramarathon.org) do not support the assumption of a large female physiological advantage in ultra-distances competitions, generally performed over multi-laps on flat roads; e.g., male vs. female record: one hour 21330 vs. 18930 m, 11.3%; 6 h 98496 vs. 85492 m, 13.2%; 12 h 177410 vs. 153600 m, 13.4%; 24 h 319614 vs. 270116 m, 15.5%, when compared to track or marathon performance. Indeed the sex-differences for 10000 m or marathon world records have fluctuated between 9% and 12% since the mid-1980s [6, 7] (the current female record for the marathon of 02:09:56, leading to a 7.8% difference when compared to the male record 02:00:35 can be seen as an outlier).

Of interest is also the observation of the mountain ultra-endurance records. In competitions held in the Alps (e.g., OCC Orsières-Champex-Chamonix, 57 km, elevation gain 3500 m, 04:42:40 vs. 05:18:21, 12.6%; CCC Courmayeur-Champex-Chamonix, 101 km, 6500 m, 09:53.02 vs. 11:40:55, 18.2%; UTMB^®^, Ultra Tour du Mont-Blanc^®^, 176 km, 10000 m, 19:49:30 vs. 22:09:38, 11.8%, https://montblanc.utmb.world/), it appears that the sex differences are also often larger than the 9–12% reported for road/track events [8]. One may question the reasons for these discrepancies and variability. They may arise from various factors, and the low participation rate of females in ultra-trail races (e.g., in 2024, 519 females (29.4%) and 1245 males (70.6%) in OCC; 484 females (21.3%) and 1785 males (78.7%) in CCC; 328 females (13.1%) and 2172 males (86.9%) in UTMB^®^) is likely one of them [5].

In this Current Opinion, we argue that, beyond potential cultural and socio-psychological reasons, three important factors specific to the mountainous environment are also influential and have to be better considered to explain sex-differences in mountain ultramarathons. The first factor is that most of the displacement during mountain ultra-trails is either uphill or downhill and this is not negligible since level and uphill running are known to be different in biomechanical and physiological factors; second, females have specific responses to terrestrial altitude-induced hypoxia; third, extreme ambient temperatures and/or change in temperature (differences between day vs. night, low vs. high altitudes) may play a role.

So, the more often you look at the scenery (beautiful in the mountains), the better the understanding of the specific responses of female athletes during ultra-trails.

## Uphill-downhill locomotion

One of the main differences between level races, such as marathons and mountain ultra-trails lies in the type of locomotor patterns. Contrary to level running in marathons (where even low-ranked athletes rarely need to walk for extended periods [9]), the alternation of uphill walking and running, along with downhill running, is crucial in mountain ultra-trails. Recent findings of our research group [10] show that sex-differences in uphill endurance performances observed in different sports (vertical race in ski mountaineering, vertical kilometre in mountain running, cycling, cross-country skiing and ultra-trail running) are larger (18–22%) than in endurance sports performed primarily on flat terrains (9–12%). Beyond the well-described factors sustaining the 9–12% sex-differences (e.g., lower maximal oxygen uptake [11], mainly due to oxygen delivery [12] and total haemoglobin mass; lower values in body size and lean mass to fat mass ratio, higher resistance to neuromuscular fatigue [13] and greater metabolic flexibility [14]), there are therefore specific factors (i.e., particularly the differences in body composition and in skeletal muscle characteristics) widening sex-differences during uphill locomotion. On shorter uphill events (e.g., sport climbing, vertical km and short climb in cycling), the sex-differences appear to be even more pronounced (28–35%), potentially due to additional factors (e.g. altered anaerobic capacity, muscle composition and/or upper body contribution in males) [15].

Of interest is that the analysis of ultra-trail running (split times for the three highest passes (col Bonhomme; col de la Seigne & Grand col Ferret of the UMTB) showed that uphill and downhill sex-differences in elite runners performance were very close (19.7% vs. 22.0%, respectively) [10]. The size of the sex differences in mountain ultra-trail is larger than in other endurance sports (e.g., ~ 9–12% in swimming, track cycling or road/track running) but lower than in power-strength sports (e.g., > 30% in weightlifting) [3]. However, there is a lower performance density (i.e., differences between the 10th and the winner’s performances) in females than in males that may impact such analysis, particularly of downhill running. Of interest is that this phenomenon of lesser female density of top performances occurs for many distances, even in non-trail running events, and it occurs even when male and female participation is about the same [16, 17]. Further research is therefore requested. Indeed, if confirmed, the reasons why the sex-differences are not smaller downhill are not fully understood; particularly, it remains unanswered if this lies in physiological factors (lower muscle mass and strength) or in perceptual factors or risk-taking strategies [18].

Overall, based on the present “uphill-downhill locomotion” hypothesis, one may predict that alternation of steep uphill and downhill sections would widen the sex-difference in performance, when compared to level running. One may speculate that this is the most important factor explaining the larger sex-difference in mountain ultra-trail performance.

### Altitude

The pulmonary system, which exhibits notable sex-based differences, is the first physiological system exposed to terrestrial hypoxia at altitude. Key anatomical differences between the sexes in the pulmonary system include smaller airway diameters and reduced lung volumes in females, even when matched for body size [19]. Consequently, females experience increased airflow resistance due to the smaller cross-sectional area of their airways, which favors turbulent airflow. This anatomical disparity results in a higher work of breathing (ẆB) in females compared to males for any given absolute minute ventilation (V̇E) [20]. Aside from comparisons at the same absolute V̇E, which represents a higher relative exercise intensity for females, it has also been shown that, at peak exercise, females have a greater metabolic cost of breathing than males when expressed as a percentage of maximal oxygen consumption [21].

While the elastic properties of the lungs and ribcage are similar between the sexes, leading to comparable elastic components of ẆB during exercise [20], the resistive component of ẆB explains the observed sex differences in total ẆB. This is likely attributable to the smaller airways in females relative to males [19, 20]. Additionally, these anatomical differences limit the capacity of females to generate high expiratory flow rates, suggesting that females may be more susceptible to expiratory flow limitation (EFL) than males [19].

However, recent studies involving large cohorts of endurance-trained athletes [21], healthy adults [22], and healthy older individuals [23] have reported no higher prevalence of EFL in females compared to males. These findings challenge earlier hypotheses and suggest that factors beyond anatomical differences may modulate the occurrence of EFL in females.

In both females and males, EFL has been proposed as an etiological mechanism underlying relative hypoventilation and subsequent exercise-induced hypoxemia (EIH) [24], which is characterized by a decrease in arterial oxygen content during exercise in normoxia. Evidence suggests that females, across all fitness levels [21] and ages [25], may be more prone to developing EIH, whereas in males this phenomenon is only observed in athletes [21]. However, this is not a universal finding since a previous study that matched males and females for height reported no sex differences in pulmonary limitations to exercise in normoxia and hypoxia [26]. The prevalence of EIH appears higher in females [21]; however, the severity of desaturation between the sexes remains to be thoroughly characterized.

Additionally, EIH is more likely to occur at submaximal exercise intensities in females [25]. Due to their smaller lungs and alveolar volumes, females exhibit a lower pulmonary diffusion capacity [27]. Overall, these factors contribute to a greater likelihood of females experiencing pulmonary limitations to exercise compared to males.

The altitude-related decrease in barometric pressure induces two phenomena that alter pulmonary function in this environment. Firstly, it reduces air density, which enhances airflow within the airways, alleviating the resistive component of ẆB for a given ventilation [28]. This reduction in resistance allows for improved expiratory flow. In this context, females may benefit more than males from the reduced air density due to their higher ẆB and potentially greater prevalence of EFL.

However, at altitude, ventilation increases via the hypoxic ventilatory response (HVR) to counteract hypoxia and ensure adequate oxygen delivery to tissues. Studies have reported mixed results regarding sex differences in HVR [29], but recent research involving a large cohort showed that females have a lower HVR compared to males, even after adjusting for body surface area [30]. As a result of the HVR, ẆB increases, and the beneficial effect of reduced air density on ẆB is often outweighed by the increased ventilation demands. Consequently, at the same absolute exercise intensity, ẆB is higher at altitude than sea level due to the increased minute ventilation [31].

This heightened ẆB at altitude increases oxygen consumption by the respiratory muscles, which may further impair performance in females. In fact, females exhibit a higher cost of breathing for a given ẆB, dedicating a greater proportion of their oxygen uptake to ventilation than males (approximately 13% in females versus 11% in males at maximal ventilation) [23]. Additionally, the increased ẆB at altitude exacerbates diaphragmatic fatigue in females. In severe acute hypoxia (5 min at FiO₂ = 0.08), inspiratory loading worsens diaphragmatic fatigue exclusively in females [32], who also fail to fully recover after 60 min following exhaustive exercise in hypoxia, whereas males exhibit complete recovery [33].

The second phenomenon associated with reduced barometric pressure at altitude is the resulting decrease in partial oxygen pressure, which inevitably reduces the alveolar-to-arterial oxygen pressure gradient. This limitation in oxygen diffusion from the lungs to the blood contributes to the development of hypoxemia [34]. In this context, the combination of a poor ventilatory response [30] and lower circulating capacity and reservoir; i.e., lower body surface area and lower normalized blood volume and hemoglobin mass (Hbmass) [35], in females may represent a greater limitation to endurance performance in hypoxia.

Moreover, knowing that females are more prone to EIH and that EIH individuals suffer from a larger altitude-related decrement in aerobic performance compared to athletes without EIH [36], one could hypothesize that overall, females may exhibit a greater performance drop due to the greater prevalence of EIH. However, a recent study reported that the well-described relationship between EIH severity and the magnitude of the altitude-related drop in performance [36] only exists in males and not in females [21].

On balance, the evidence supports the view that the female pulmonary system during exercise, and consequently their aerobic performance, may be more negatively impacted by a hypoxic environment. Notably, the mechanisms involved are proportional to elevation, and mountain ultramarathons can take place at a wide range of altitudes. Indeed, the potential sex-related differences due to pulmonary limitations will have a greater impact at higher elevations and be more limited at lower ones. Moreover, some existing evidence from studies conducted in normoxia comparing sex differences during standard exercise reports greater pulmonary limitation in females. Further studies on sex-related differences in pulmonary limitation during exercise in hypoxia and especially during ultra endurance exercise in hypoxia, are needed to improve our understanding of these potential sex differences. 

## Ambient temperature

Mountain ultra-trails take place in mountain environments where participants are frequently exposed to freezing temperatures, either as an isolated stressor or in combination with the challenges of high altitude [37]. However, the influence of ambient temperature on the widening of sex differences in mountain ultra-trail performance warrants further investigation. Despite the insulation added by their increased subcutaneous fat layer [38], females have a greater body surface area-to-mass ratio, which contributes to higher heat dissipation, as well as lower muscle mass, limiting their overall capacity for thermogenic heat production [5, 39]. Additionally, previous studies have shown that the vasoconstrictor response during cooling is reset to a higher body temperature in females [40], particularly during the luteal phase of the menstrual cycle, when resting body temperature increases by ≈ 0.3 °C [41]. This adjustment may result in earlier exhaustion of maximal cutaneous vasoconstriction capacity, explaining why females often rely on shivering heat production sooner than males [42]. Interestingly, although lower muscle mass reduces the total heat generated through shivering, females’ natural tendency to favor fat over carbohydrate oxidation may provide an advantage during prolonged cold exposure by enhancing shivering endurance [43]. Finally, brown adipose tissue (BAT) volume and metabolic activity are similar between the sexes when normalized for body mass index, body surface area, or lean mass [40], but as for the vasoconstrictor response, BAT thermogenesis begins at higher temperatures in females [44]. Adding complexity to this scenario, most of the described responses have been studied at rest, leaving uncertainty about how higher adiposity and lower muscle mass might mitigate sex differences in thermoregulation during exercise by reducing excessive heat loss when blood flow to muscles and the periphery increases [40].

Besides physiological responses, behavioral thermoregulation (e.g., dressing or undressing) represents another critical defense against core cooling, and females have been shown to exhibit greater thermal awareness and rely more heavily on thermoregulatory behaviors than males [44]. Despite this being helpful in avoiding dangerous decreases in core temperature, Raynaud’s phenomenon (characterized by hyper-responsiveness of cutaneous vasoconstriction in the fingers and toes during cold exposure) is more prevalent in females [45], potentially increasing their risk of race withdrawal due to severe cold injuries involving the extremities.

As noted earlier, mountain ultra-trails often involve simultaneous exposure to cold and high altitudes. In such scenarios, competition arises between maintaining oxygen delivery to distinct vascular beds (hypoxia-induced vasodilation) and cold-induced vasoconstriction for heat conservation [46]. This interplay can result in elevated skin temperature and greater heat dissipation [47]. Evidence suggests that young females exhibit greater compensatory peripheral vasodilation at rest and increased relative vasodilation (forearm vascular conductance normalized to volume) during exercise in hypoxia [48]. Consequently, in combined stressor exposure, females may experience a faster decline in core temperature than in the cold alone, further expediting shivering and non-shivering thermogenesis compared to males.

Heat exposure during mountain ultra-trails is much less frequent but still eventually possible, especially when considering races in specific earth areas (e.g., Badwater 135, Death Valley) or lower altitude sections of competitions held in the summer period (e.g., UTMB^®^). Compared to cold exposure, less muscle mass and a greater body surface area-to-mass ratio in females should be advantageous in the heat [49]. Despite this, previous studies showed impaired vasomotor and sudomotor responses in females, but these studies included the intrinsic error of comparing the two sexes during exercises that did not evoke equivalent heat loss requirements [50]. Once this aspect is considered, the onset threshold and thermosensitivity of cutaneous vasodilatation have been shown to be similar between males and females, but still, lower sudomotor activity, as well as a lower thermosensitivity of the response in the latter have been detected [51]. However, the extent to which this is influenced by sex hormones or simply reflects an adaptation of thermo-effector responses to the subjects’ morphological characteristics remains debated. In fact, a recent study demonstrated similar responses between the sexes after accounting for differences in mean body temperature and morphology [52]. Nevertheless, females appear to be at a thermoregulatory disadvantage in dry heat conditions, whereas this disadvantage may be diminished in humid environments, where the heat dissipation mechanism via evaporation is not constrained by individual factors but rather by external environmental conditions [49].

In summary, the ambient temperature hypothesis for the widened sex gap in mountain ultra-trail performance is based on the higher prevalence of extreme environmental conditions, particularly cold temperatures, in these races compared to most other endurance sports. Additionally, the simultaneous exposure to cold temperatures and high altitudes may further exacerbate this gap compared to races held in cold conditions at sea level. Finally, the repeated alternation between cold and hot-dry environments, as athletes move between higher and lower altitudes multiple times during the race, may pose a greater thermoregulatory challenge for females. Future research should explore the interaction of cold, hypoxia, and sex during exercise to develop targeted strategies that minimize the sex performance gap and ensure the safety of all mountain ultra-trail participants.

## Conclusion

**Fig. 1:**
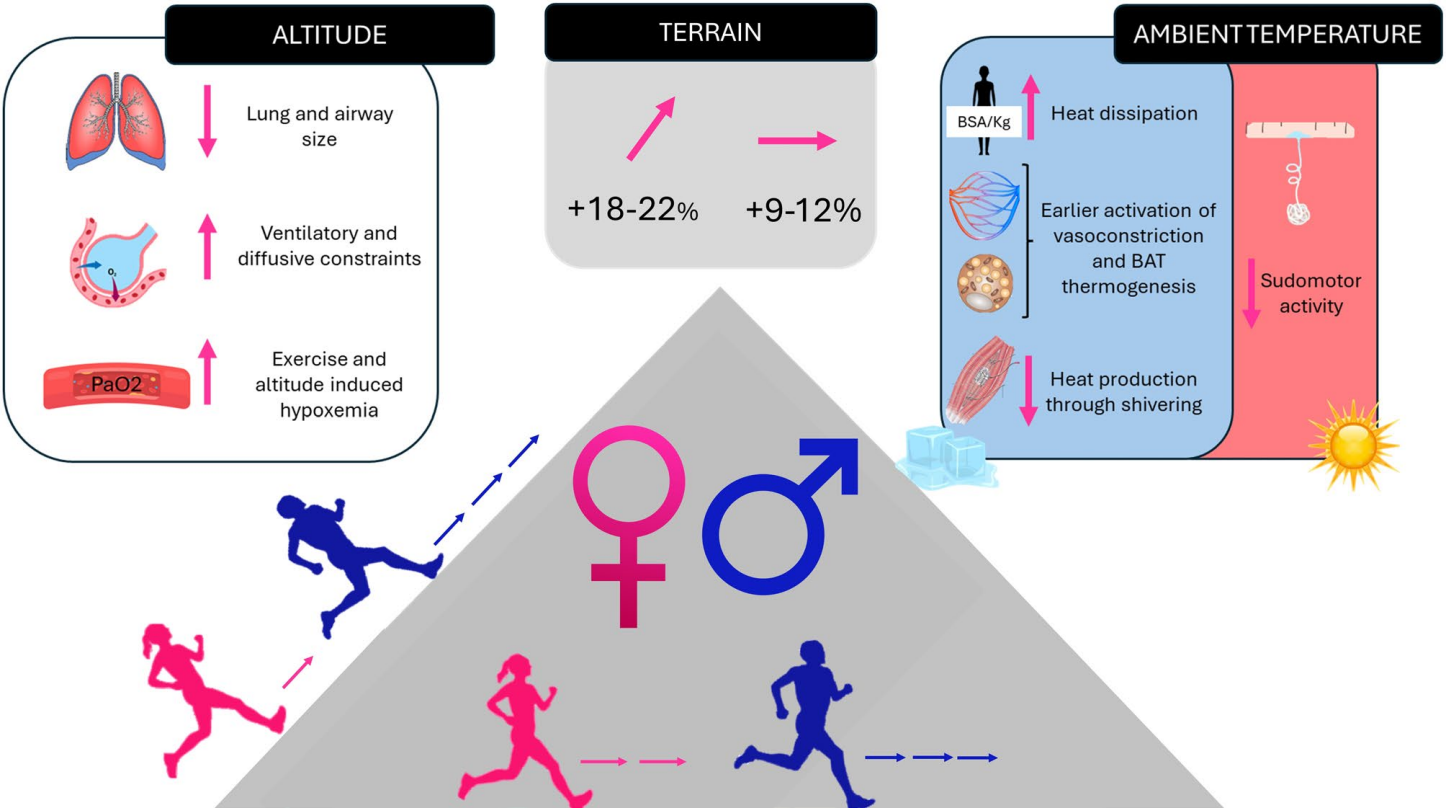
Schematic representation of the three main factors widening sex-differences in ultra-trail performances when performed in mountains: altitude, ambient temperature, and uphill-downhill locomotion. PaO_2_: Partial Pressure of Oxygen in Arterial Blood; BSA: Body Surface Area; BAT: Brown Adipose Tissue

In this article, we pointed out that the sex differences in mountain ultra-trail performances are largely influenced by specific factors related to the mountainous conditions, i.e., uphill locomotion, altitude, and extreme ambient temperatures. However, despite the above-mentioned female disadvantages in cold environment and altitude (Fig. 1), it is also noteworthy to point out that female endurance athletes are capable of extraordinary achievements, outperforming male counterparts in extreme cold environments and altitude [53] such as in Antarctica.

There are likely other factors implicated, such as a more conservative and therefore effective pacing in female runners [54]. Since in challenging conditions such as high-altitude, the risk-taking strategy of the females is safer than those of their male counterparts, one may speculate that the pacing strategy between females and males may differ to a larger extent in mountain ultra-trails than in low-altitude events. However, further research is requested to validate this hypothesis.

## Data Availability

Not applicable.

## Abbreviations

BAT: Brown Adipose Tissue
CCC: Courmayeur Champex Chamonix
EFL: Expiratory Flow limitation
EIH: Exercise-Induced Hypoxemia
FiO2: Fraction of Inspired Oxygen
Hbmass: Hemoglobin Mass
HVR: Hypoxic Ventilatory Response
OCC: Orsière Champex Chamonix
UTMB®: Ultra Tour du Mont-Blanc
V̇E: Minute Ventilation
ẆB: Work of Breathing
